# Vitamin D and Uterine Fibroids—Review of the Literature and Novel Concepts

**DOI:** 10.3390/ijms19072051

**Published:** 2018-07-14

**Authors:** Michał Ciebiera, Marta Włodarczyk, Magdalena Ciebiera, Kornelia Zaręba, Krzysztof Łukaszuk, Grzegorz Jakiel

**Affiliations:** 1 Department of Obstetrics and Gynecology, Centre of Postgraduate Medical Education, 00-416 Warsaw, Poland; kornelia3@poczta.onet.pl (K.Z.); grzegorz.jakiel1@o2.pl (G.J.); 2 Department of Biochemistry and Pharmacogenomics, Faculty of Pharmacy with Division of Laboratory Medicine, Medical University of Warsaw, 02-097 Warsaw, Poland; mdwlodarczyk@gmail.com; 3 Center for Preclinical Research, Medical University of Warsaw, 02-097 Warsaw, Poland; 4 Students’ Scientific Association at the Department of Obstetrics and Gynecology, Medical University of Warsaw, 02-015 Warsaw, Poland; mciebiera93@gmail.com; 5 Department of Obstetrics and Gynecological Nursing, Faculty of Health Sciences, Medical University of Gdansk, 80-210 Gdansk, Poland; luka@gumed.edu.pl; 6 INVICTA Fertility and Reproductive Center, 80-172 Gdansk, Poland

**Keywords:** uterine fibroid, leiomyoma, vitamin D, vitamin D receptor, vitamin D analogs

## Abstract

This article provides a detailed review of current knowledge on the role of vitamin D and its receptor in the biology and management of uterine fibroids (UFs). Authors present ideas for future steps in this area. A literature search was conducted in PubMed using the following key words: “uterine fibroid” and “vitamin D”. The results of the available studies, published in English from January 2002 up to April 2018, have been discussed. Vitamin D is a group of steroid compounds with a powerful impact on many parts of the human body. This vitamin is believed to regulate cell proliferation and differentiation, inhibit angiogenesis, and stimulate apoptosis. Nowadays, hypovitaminosis D is believed to be a major risk factor in the development of UFs. In many studies vitamin D appears to be a powerful factor against UFs, resulting in inhibition of tumor cell division and a significant reduction in its size, however, the exact role of this compound and its receptor in the pathophysiology of UFs is not fully understood. According to available studies, vitamin D and its analogs seem to be promising, effective, and low-cost compounds in the management of UFs and their clinical symptoms, and the anti-tumor activities of vitamin D play an important role in UF biology. The synergy between vitamin D and selected anti-UF drugs is a very interesting issue which requires further research. Further studies about the biological effect of vitamin D on UF biology are essential. Vitamin D preparations (alone or as a co-drugs) could become new tools in the fight with UFs, with the additional beneficial pleiotropic effect.

## 1. Introduction

Uterine fibroids (UFs) are monoclonal, benign tumors which arise from the smooth muscle cells of the uterus and are predominantly located in the pelvis. They constitute one of the most common pathologies of the female genital tract. UFs occur in 5–70% of all women [1,2,3,4], and develop in selected populations, with age and ethnicity as the main risk factors [3,4]. UFs are more prevalent among reproductive aged women and are not observed in pre-pubescent girls, indicating that tumor origin depends on hormonal changes [1,2,5]. Extensive research has identified several factors connected with higher UF occurrence, but data are inconsistent and conflicting [3,6]. In a recently published systematic review, Stewart et al., underlined factors which increase the incidence of UF, and these are: black race, elevated body mass index (BMI), age, premenopausal status, hypertension, positive family history, time elapsed since last labor, consumption of food additives, and soybean milk [3]. 

Apart from the abovementioned risk factors, recent studies suggested that hypovitaminosis D plays a role in UF development [6,7,8,9]. The Study of Environment Lifestyle and Fibroids (SELF) was performed to describe the actual contribution of hypovitaminosis D and other factors to the development of fibroids [10]. Most of the research on this field focuses on Afro-American women, who are at an increased risk for UF occurrence [9,11]. Among Afro-American women, vitamin D deficiency because of higher melanin concentrations results in decreased serum vitamin D levels [7] and reduced expression of the vitamin D receptor (VDR) in the adjacent myometrium [12], compared to white women [13]. 

UF tumors vary greatly in size, location, and symptoms [2,14]. Most tumors are largely asymptomatic, but they may also cause a wide range of severe and chronic symptoms [3,4,15] in approximately one-quarter to one-third of the affected women [3,14]. The most common symptoms include abnormal and excessive uterine bleeding, secondary iron deficiency anemia, abdominal and pelvic pain, gastric disorders like bloating and constipation, voiding symptoms, infertility and obstetric pathologies (including miscarriage and premature labor) [1,2,14,16,17].

Despite their unquestionable effect on the quality of patient life (QoL), UF-related QoL is very often marginalized [18,19]. Also, the financial burden on the healthcare budget is considerable, including the costs of preoperative diagnosis, surgical treatment, hospitalization time, work absenteeism, medicines, salaries of the medical workers, and the costs of control visits [4,20,21]. The annual direct and indirect costs of UFs in the United States have been estimated at approximately $4.1–9.4 billion and $1.6–17.2 billion, respectively [4,20,21]. 

Clinically symptomatic UFs are most often treated with surgery [2,22]. Various types of surgical methods are available, both open and endoscopic (hysterectomies, myomectomies, hysteroscopic resections) [22,23]. UFs are the leading reason for the hysterectomy [21,24]. The optimal treatment should reduce blood loss and tumor burden, while preserving fertility [25]. Women who wish to retain their uterus can be offered with less invasive methods [22,23,26]. Nevertheless, many of them will require a re-intervention in the future [27]. Due to the benign nature of the tumors, the first-line treatment should result in lowest morbidity and risk of adverse effects [23,28]. Multiple evidence has suggested progesterone to be the major initiator of UF development and stimulator of their further growth [29,30]. Thus, it is not surprising that ulipristal acetate (UPA), a selective progesterone receptor modulator (SPRM), has become one of the most popular pharmacological treatments of UFs [23,28,31]. Due to its effectiveness, UPA is administered as first-line therapy to prepare UFs for surgery. In some cases, if the effect is satisfying, UPA can be use as the only treatment [31]. However, UPA is not inexpensive, nor is it a substance which can be widely used in prevention for a long time [32]. Also, the European Medicines Agency (EMA) has recently issued warning about the risk of liver failure after UPA use [33], but research is still ongoing and there is not enough information at present about the matter.

The origin of UFs is multifactorial and that is why there are no specific methods of prevention at present [34,35]. Numerous attempts have been made to create inexpensive, safe, and effective methods of prophylaxis but they are still in the early stages [34,35]. In light of this, vitamin D, which plays one of the major roles in UF biology, might be the answer [13]. 

**Figure 1:**
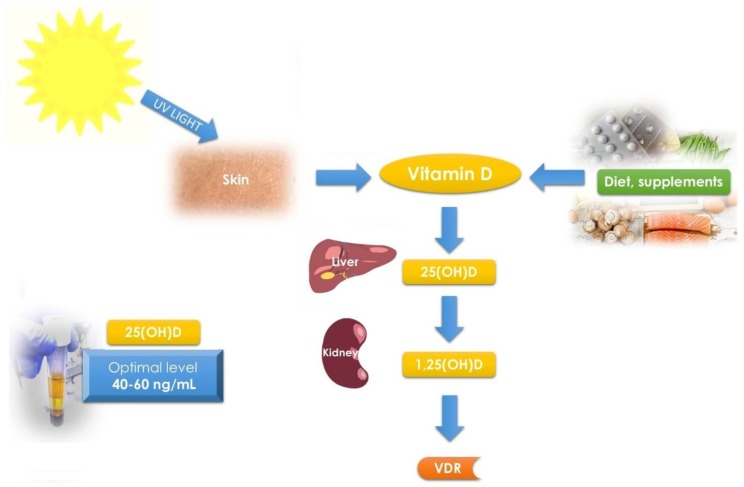
Vitamin D metabolism and schematic pathways. Diet, supplements and sunlight are the major source of vitamin D in humans. Vitamin D is synthetized in skin from 7-dehydrocholesterol. In further steps liver converts it to 25(OH)D and then kidney to 1,25(OH)D. Optimal vitamin D serum levels were described as 25(OH)D of 40–60 ng/mL [39].

Vitamin D is a name for a group of steroid compounds, soluble in fats, which exert powerful effects on the human body, and whose receptors are found in various organs [36,37], including the myometrium and UF tumor tissue [38] (Figure 1). 

Vitamin D takes part in cell cycle regulation and cell differentiation, and it also has anti-angiogenic activities [40]. Vitamin D deficiency is an important risk factor in the development process of UFs [6,7,8,9,41]. There are several ideas about the use of vitamin D in UF prevention or as a long-term treatment [13,34,42], but ongoing clinical trials in this area remain scarce.

Despite the accumulating data on UFs, information about the involvement of vitamin D in their pathophysiology is limited. Thus, we present an up-to-date review about the role of vitamin D in UF-associated problems, as well as our ideas for future steps.

## 2. Materials and Methods

This article presents an up-to-date review of the publications regarding the current role of vitamin D and its receptor in the pathophysiology and management of uterine fibroids. A literature search was conducted in PubMed using the following key words: “uterine fibroid” and “vitamin D”. During our search, we combined the key words into a pair, and found 45 publications. The aim of the review was to evaluate the current state of knowledge about the role of vitamin D in uterine fibroid biology and management. The results of the available studies, published in English from January 2002 up to April 2018, have been discussed. Additional important articles and reviews were considered, when relevant.

## 3. Discussion

### 3.1. Uterine Fibroid Biology—Overview

Genetic studies have proven UFs to be monoclonal hormone-dependent tumors [30,43]. Tumor development begins with the creation of a pathologically changed and transformed primary myometrial cell. Subsequently, all secondary cells divide, making the tumor grow further [44]. Modified cells need proper stimulation in order to divide and produce the extracellular matrix (ECM) [45,46]. The mechanisms controlling the growth of UFs are complex and still not well-recognized [47]. Abnormal and excessive ECM production is a major factor in UF growth [46,47]. 

The main hormones which simulate UFs development and growth are estrogen [48] and progesterone [29,30,49]. Estrogen [48] and progesterone [49] induce UF formation and growth, affecting them directly and indirectly through various growth factors [50,51]. Numerous experts consider progesterone to be the main steroid initiating uterine muscle differentiation, and its subsequent abnormal growth [30,43,49,52,53,54]. The effect of progesterone on UF growth has been confirmed by the wide use of its antagonists (SPRMs) in the treatment of UFs [31,55,56]. Estrogens, which play a smaller role in the UF pathophysiology, prepare the tumor to be stimulated by progesterone by upregulating its receptors [48,57]. 

UFs are greatly affected by genetic abnormalities [58,59,60]. Since the discovery made by Makinen et al., in 2011 [58], UF genetics has made great advances. Specific mutations within the MED12 gene which encode the mediator complex subunit 12 (MED12) are detected in almost 80% of the UF samples [58,59]. So far, no mutations have been found in MED12 in the healthy myometrium of the women studied [60].

### 3.2. Vitamin D and Its Receptor—Overview

Vitamin D is a group of steroid compounds which have a powerful impact on many parts of the human body, including the musculoskeletal, nervous and immune systems, as well as the genital tract [36,37,61]. The main activity of vitamin D concerns the control of calcium-phosphate balance as well as the correct structure and function of the skeleton [36,62]. Although it is traditionally included in vitamins, vitamin D also fulfills the requirements to be classified as a hormone [63,64]. Vitamin D can occur in several forms—vitamin D1, or calciferol (most often found in fish oils), vitamin D2—ergocalciferol (found in plants) and vitamin D3—cholecalciferol (produced in the skin) [65,66]. Vitamin D is converted to 25-hydroxyvitamin D [25(OH)D] by the 25 α-hydroxylase enzyme in the liver, and after that it is hydroxylated in the kidneys to 1,25-dihydroxyvitamin D [1,25(OH)D] [64]. The most active form of this vitamin—1,25(OH)D—presents its activity in almost every tissue in human body [13]. 

Vitamin D is carried by a specific transportation protein—Vitamin D-binding protein (VDBP)—which belongs to the albumin gene family [67]. This protein transports various forms of vitamin D, including ergocalciferol, cholecalciferol, calcifediol and calcitriol, between the skin, liver and kidneys, and then on to various target tissues [63]. According to Yao et al., similar levels of VDBP were observed in the population of Euro-American and Afro-American women [68]. There are some studies which have already demonstrated the usefulness of VDBP in clinical diagnosis. It might be used as a biomarker for selected diseases, for example, breast cancer [69,70]. Also, in their study from 2012, Lin et al., indicated that VDBP can be used as a potential marker for UFs [70].

Vitamin D is believed to regulate cell proliferation and differentiation, inhibit angiogenesis, and stimulate apoptosis [36,37,61]. Vitamin D works by a specific type of receptor—VDR. It is a mediator of the pleiotropic effect of this vitamin [71]. Vitamin D mediates its metabolic functions through steroid transcriptional mechanisms [64,71]. This vitamin can modulate the expression of various genes in a tissue-specific manner, and then can lead to the inhibition of cell proliferation, differentiation, and apoptosis. These processes can take part in the inhibition of neoplastic transformation as well as tumor growth, such as in UF [13,72].

Endogenous vitamin D production is limited by factors such as geographical location, environmental and individual characteristics (e.g., latitude, season, weather conditions, clothing), as well as the use of sunscreens and other cosmetics [73,74]. Abnormal supplementation and insufficient exposure to solar radiation due to spending the majority of time indoors are believed to be the main reasons for vitamin D deficiency in white female population [73]. People with dark skin, especially black, must spend 5 to 10 times more time outside to produce the same portion of vitamin D as compared to people with fair complexion [73,75], which is the reason why, for example, Afro-Americans are more likely to have low levels of vitamin D [76].

Vitamin D levels defined as “deficient” are the subject of much heated debate among the experts [37,39,77]. According to the Endocrine Society Practice Guidelines on vitamin D status, “deficiency” is defined as 25(OH)D level of <20 ng/mL, insufficiency as 21–29 ng/mL, and sufficiency as at least 30 ng/mL (for the best overall musculoskeletal effect) [78,79]. The actual guidelines suggest a preferred range from 40 to 60 ng/mL when focusing on the pleiotropic effect of vitamin D [39,61,80] (Figure 1). 

### 3.3. Vitamin D in Uterine Fibroid Biology

Vitamin D is believed to reduce the risk of chronic illnesses and neoplasms [37,61]. According to the review by Grant, the available scientific evidence supports the notion of vitamin D supplementation as a cancer prevention method [81].

Decreased serum vitamin D levels have been already confirmed in several gynecological and obstetrical pathologies, such as infertility or polycystic ovary syndrome [82,83,84,85,86]. Vitamin D is also known to affect cycle regularity through its effect on hormones such as insulin or androgens. Various studies have confirmed that lower serum 25(OH)D levels were associated with irregular menstrual cycles [87]. Vitamin D may also influence the ovarian reserve and is inversely related to FSH level, as was demonstrated by Jukic et al. [88]. 

Recent studies have identified abnormal concentrations of vitamin D as important players in the etiology of UFs [6,7,8,9,38]. Nowadays, vitamin D deficiency is believed to be also a major risk factor in the development of UFs. Mean 25(OH)D serum levels are significantly lower in UF-positive women as compared to UF-negative controls [6,7,8]. These findings were also confirmed in Turkish [89] and African-American populations, who are more likely to present both with vitamin D deficiency and presence of UFs [90]. Cultural and environmental differences might play a role in the UF development as well [91], Oskovi Kaplan et al., suggested that traditional clothing style (covering the body), low education or being a housewife are also risk factors for vitamin D deficiency which, at some point in life, might result in UF [89]. Recently, theories about the vital role of vitamin D in the pathogenesis of UFs, and research into the effects of vitamin D on UFs, have gained new momentum. Vitamin D has become one of the key elements of modern theory of UF pathogenesis [6,13]. Epidemiological studies continue to emphasize the role of vitamin D deficiency in the development of UFs. One of the most recently published studies on these correlations was published in 2015. Mitro et al., in their study on 3600 women who took part in the National Health and Nutrition Examination Survey (NHANES) between 2001 and 2006, found no association between low vitamin D levels and the appearance of UFs within the entire population [92]. Interestingly, taking into account only the white population, the decreased serum concentration of vitamin D was a risk factor for UFs, but no such correlation was observed in black women [92]. Thus, larger studies are still necessary to better understand the biology of UF.

**Figure 2:**
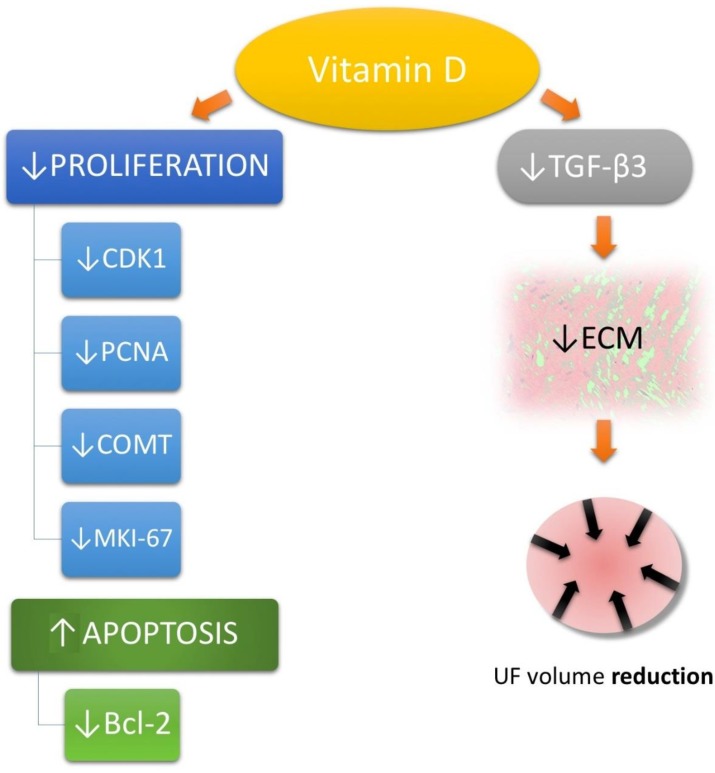
Vitamin D influence on uterine fibroid (UF) pathophysiological pathways. Cyclin-dependent kinase 1 (CDK1), proliferating cell nuclear antigen (PCNA), catechol-*O*-methyltransferase (COMT), Bcl-2 protein, proliferation marker protein Ki-67 (MKI-67), extracellular matrix (ECM), transforming growth factor beta 3 (TGF-β3), uterine fibroid (UF).

The results of the first study conducted to better understand the effect of vitamin D on the growth of UF were published by Blauer et al. [38]. The research was carried out in 2009 and showed the relationship between 1,25(OH)D levels and the growth of UF cells (samples were obtained from women who underwent hysterectomy) [38]. Inhibition of their growth was correlated with vitamin D concentration and increased with increasing vitamin D concentration [38]. In another study, performed later by Sharan et al., 1,25(OH)D caused in vitro inhibition of proliferation of immortal UF cells [93] (Figure 2). 

Their findings were unambiguous: proliferating cell nuclear antigen (PCNA)—known as a molecular marker for proliferation [94], cyclin-dependent kinase 1 (CDK1)—a protein kinase complex known as M-phase promoting factor [95], Bcl-2—considered an important anti-apoptotic protein [96], and catechol-*O*-methyltransferase (COMT)—which is involved in estrogen metabolism [97], were all highly affected by vitamin D compounds [93]. In the same year, Halder et al., published a study which showed the effect of vitamin D3 on the transforming growth factor beta (TGF-β) pathway. In their study, TGF-β3 was inhibited by increased concentrations of vitamin D [98]. Their results further confirmed our belief that research about the role of vitamin D in the UF biology is the right direction because TGF-β is considered to be one of the most relevant factors in the pathogenesis of fibrosis-associated diseases [46], while TGF-β3 is one of the most important TGF-β isoforms in UF biology [99,100,101]. Its increased serum levels constitute a risk factor for UF incidence [6]. TGF-β3 slows the degradation of ECM [99,102], and plays a vital role in its overproduction by stimulating the expression of selected ECM molecules, such as proteoglycans and proteins [46,103] (Figure 2). In subsequent studies, which have been carried out on animal models (Eker rats), therapeutic doses of vitamin D were found to significantly reduce the size of UFs by suppressing genes responsible for cell growth and cell division, antiapoptotic genes and genes encoding estrogen and progesterone receptors [104,105]. In animal models, vitamin D presented great effect on molecular genetics by suppressing cell growth and proliferation-related genes (e.g., Pcna, Cdk1, Cdk2, Cdk4), antiapoptotic genes (Bcl2 and Bcl2-like1), and estrogen and progesterone receptors [104]. In the same study, immunohistochemical staining revealed decreased expression of additional markers of proliferation (PCNA and MKI-67) [104]. 

According to an interesting study by Al-Hendy et al., 1,25(OH)D functions as a potent antiestrogenic and antiprogesteronic agent [106]. These authors observed an inverse correlation between the up-regulated estrogen and progesterone receptors and VDR expression in UFs. In the same study, treatment with active vitamin D significantly decreased the levels of estrogen and progesterone receptors [106]. Steroid hormones and their receptors are crucial in UF biology. For example, due to their influence on Wnt/β-catenin and TGF-β pathways [101]. In many tumors, including UFs, cytokines and growth factors play the key role in inflammation and regulation of cell division [40,50,107]. Recently, the elevated expression of activin A and its effect on inflammation and fibrosis have been thoroughly documented as well (e.g., in UFs) [108]. These factors may be also responsible for UF-associated symptoms such as infertility or pain [40], for example, TGF-β (especially the TGF-β3 isoform) which is one of the most important factors in the development and growth and UFs and the related problems [50,98,101]. What is already known is that UFs regulate and stimulate the accumulation of ECM, with TGF-β as the mediator [57,101,109]. Overexpressed TGF-β induces ECM overproduction by stimulating the expression of type I collagen, proteoglycans, and other ECM compounds, what in turn results in abnormal ECM accumulation [101,103,110]. VDR activation by its ligand results in reduced inflammation and fibrosis [34,111]. Owing to the studies by Halder et al., it was possible to prove that a surprisingly beneficial effect in UF growth reduction can be obtained under the influence of vitamin D [98,104]. In their papers, authors concluded that 1,25(OH)D reduces TGF-β3-related gene expression and 1,25(OH)D treatment reducing growth of uterine leiomyoma tumor and inhibition proliferation of leiomyoma cells [98,104]. In 2016, Al-Hendy et al., proved that vitamin D administration reduced the levels of Wnt4 and β-catenin in UF cell cultures [112]. These authors suggested that vitamin D might function as an inhibitor of Wnt4/β-catenin and mechanistic target of rapamycin (mTOR—kinase which regulates cell growth, cell proliferation and cell motility) signaling pathways [112]. In the same study, the authors made an interesting observation about the gene encoding flap structure-specific endonuclease 1 (FEN1) [112,113]. FEN1 is an enzyme involved in DNA damage repair, which was found to be overexpressed in the majority of cancers [114]. When FEN1 is overexpressed, the highly inaccurate DNA repair pathway may be favored, causing a great risk of potential mutation and increased risk of cancer [115]. Al-Hendy et al., tested the effect of vitamin D on the FEN1 protein expression in human UF and found that vitamin to effectively suppress FEN1 in a concentration-dependent manner. They concluded that this was yet another clue indicating the potential use of vitamin D in UF treatment [112].

There are other ways to explain the beneficial effects of vitamin D on UFs. Matrix metalloproteinases (MMPs) are calcium-dependent zinc-containing endopeptidases which play a role in continuous ECM rebuild [116]. MMPs are capable of degrading all kinds of ECM proteins [116]. MMPs enzymes are regulated by tissue inhibitors of metalloproteinases (TIMPs) [117]. In 2013, Halder et al., demonstrated that vitamin D increased TIMP expression in the uterine myometrium. That study revealed that vitamin D plays an important regulatory role in the expression and activities of MMP-2 and MMP-9 [118]. 

Potential antitumor properties of vitamin D have been covered by other studies, such as on mesenchymal multipotent cells [119]. Many of the pathways in the mesenchymal multipotent cells were found to be similar to those described in UFs. Artaza et al., observed increased expression and nuclear translocation of VDR, decreased expression of TGF-β, collagen I, III and increased expression of bone morphogenic protein 7 (BMP-7) and MMP-8 [119].

### 3.4. Vitamin D Receptor and Uterine Fibroids—Gene Polymorphisms

Data on single nucleotide polymorphisms (SNPs) in UFs are more scarce than other genetic findings. 

In 2014, a group of researchers began their work on the study of SNP gene polymorphisms and its correlation with UFs occurrence [120]. Preliminary studies have shown that SNPs related to the metabolism of vitamin D and skin color are associated with the presence of UFs in black women [120]. Among studied SNPs rs12800438 near *DHCR7* and rs6058017 in *ASIP* gene are implicated in vitamin D synthesis in the skin [120]. The relationship between UF and rs739837 and rs886441 polymorphisms in the nuclear hormone receptor for vitamin D has been described [120]. The study by Shahbazi et al. supports the hypothesis that UFs are associated with the VDR rs2228570 polymorphism—correlation between VDR TT genotype and UF occurrence risk [121]. More recently (2018), Yilmaz et al. demonstrated that the presence of the rs2228570 CC genotype may be a risk-reducing factor and the T allele may be a potential risk factor for the development of UFs, which is consistent with the findings of Shahbazi [122]. Both studies had limitations: small sample size and closed populations and their results need to be confirmed on larger populations [120].

### 3.5. Vitamin D—Potential Uterine Fibroid Prophylaxis or Treatment Method

#### 3.5.1. Vitamin D—Optimal Levels and Supplementation against Uterine Fibroids

The least studied factors which affect the risk for UF occurrence are related to lifestyle, diet, nutrition, or place of residence. Especially nutrition and diet can be the gateway to effective prevention of UFs [34,123,124]. As the new guidelines from 2018 defined optimal concentration of vitamin D at 40–60 ng/mL (Figure 1) [39], vitamin D supplementation and sunlight exposure can be the two main clues for UF prevention [125]. In cases of deficiency, vitamin D can be raised to the correct level by taking a supplemental dose—7000 international units (IU)/day or 50,000 IU/week, depending on patient choice [39]. Chronic administration of high doses of vitamin D may lead to its toxic effects, manifested by severe hypercalcemia and functional hypoparathyroidism, resulting in fractures and osteoarticular pain [126]. It seems that the undesirable effects of vitamin D can be bypassed by short-term high-dose therapies instead of chronic administration [37]. 

In 2016, in a study performed by Ciavattini et al., 53 women received vitamin D supplementation [127], which restored correct vitamin D serum concentrations in women with small burden UFs (<50 mm in diameter and less than 4 tumors). In these women, treatment with vitamin D reduced disease progression. To the best of our knowledge, this is the first study showing beneficial results of vitamin D use in UF management in humans [127].

In our opinion, vitamin D seems to offer a promising, effective, and low-cost prevention or treatment of UFs and their clinical symptoms. Should further findings be positive, vitamin D supplements/drugs could become a new weapon in the battle against UFs, with the additional beneficial pleiotropic effect. Furthermore, skeletal and extra-skeletal advantages support the use of vitamin D as a prophylactic agent in high-risk or UF-positive women [34]. 

#### 3.5.2. The Use of Paricalcitol in Uterine Fibroid Management

Potential adverse effects of chronic or high-dose vitamin D treatment might be bypassed by using vitamin D analogs [34,105]. The experimental trials performed in animal models provided evidence that VDR agonists have a therapeutic potential in chronic inflammatory diseases and cancer [128]. Interestingly, VDR agonists show agonistic, partial agonistic, or antagonistic activity, depending upon the structure of their side chains [129]. Paricalcitol is a selective vitamin D analog, a VDR activator used mostly in the treatment of secondary hyperparathyroidism [111,130]. These analogs are already present on the market for different indications. However, subsequent studies suggest that they may also have a beneficial anti-proliferative effect on UFs [105,131]. 

Most of the activities which may be useful in the treatment of UF can be explained on the basis of the observations obtained in nephrological models [132,133]. In kidneys, paricalcitol presented an immunomodulatory effect which can cause limited ECM thickening and may slow down angiogenesis [111,134]. In the same model, paricalcitol interfered with TGF-β1 activation of the TGF-β receptor 1 [135]. The observation is intriguing due to the presented anti-inflammatory and anti-fibrotic properties of this vitamin D analog [135]. According to the data obtained, paricalcitol has an influence on Wnt/β-catenin signaling as well as on NF-κB, which results in decreased expression of ECM [132,133]. The same observations about Wnt/β-catenin signaling are present in UFs, where increased secretion of Wnt ligands under the influence of steroid hormones leads to excessive production of different TGF-β and ECM isoforms, as well as enhanced proliferation of UF stem cells [30,57,101]. In our opinion, we can transfer data about fibrosis to UFs models to a certain extent, because these tumors consist largely of ECM with embedded cells, and excessive ECM production is considered to be one of the key mechanisms of UF formation [101]. 

### 3.6. Future Concepts in the Area of Uterine Fibroids and Vitamin D

Recent attempts to create a cheap, safe and effective drug targeted at the prevention and treatment of UFs remain in the very early stages, and it is not known whether they will succeed. Vitamin D is a natural supplement which may prevent UF development and growth, and undoubtedly deserves further investigation [136,137].

**Figure 3:**
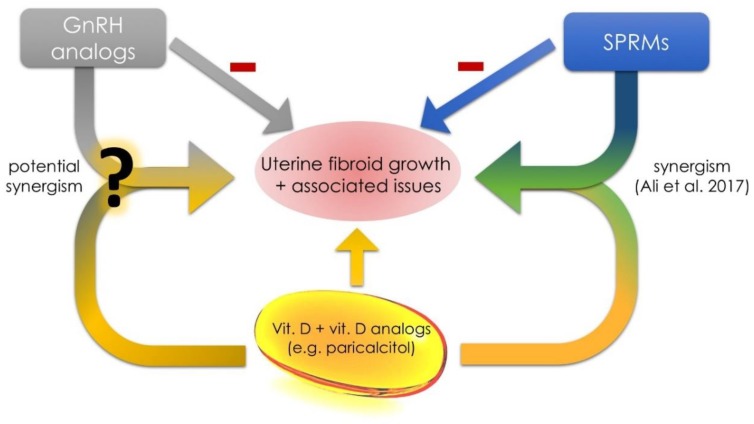
Vitamin D, GnRH analogs and SPRMs. According to Ali et al. there is a potential synergism between vitamin D and ulipristal acetate [140]. New data about between vitamin D, vitamin D analogs and other mostly used drugs in UF therapy is necessary to find other synergisms. Gonadotropin releasing hormone (GnRH), selective progesterone receptor modulator (SPRM).

Vitamin D seems to be a promising, safe and low-cost agent in the prevention or treatment of UFs. Further reports are necessary to prove the efficacy of vitamin D supplementation in women [138]. In cases of further positive observations and effects in randomized trials, vitamin D preparations could become a new generation of anti-UF drugs [42,139] (Figure 3). 

There is evidence supporting the beneficial action of vitamin D supplementation in women with small UFs [127], but further extensive studies are needed to fully understand the exact role of vitamin D in UF biology. Lack of randomized controlled trials on vitamin D use in the prophylaxis or treatment of UFs remains a significant problem. In our opinion, the main reason for that is the lack of unified cut-off thresholds for vitamin D deficiency—they differ in different countries [39]. The consensus in this area can bring tangible benefits to women with UFs. Currently, high-risk patients, those with positive history of UFs, Afro-Americans and those with elevated BMI, should be screened and offered supplementation, if necessary [6]. According to Ali et al., women who would benefit from this management include also these with early menarche, nulliparous, and aged <40 [141].

An additional aspect that should be implemented when constructing subsequent clinical trials and determining recommendations for vitamin D supplementation is an individually differentiated response to vitamin supplementation [142]. According to available data, up to 25% of humanity can be considered as vitamin D low responders (slow response after standard supplementation doses) [142]. In the matter of UFs, the fact of a different response to supplementation doses may be of great importance, both to the effectiveness of treatment and also for economic reasons [20]. For example, similar doses of vitamin D in a high responders group may result in high raises of 25(OH)D serum levels and subsequent retention of tumor growth, whereas low responders will only gain a very low 25(OH)D serum level raise and small pleiotropic effect [142]. It seems, therefore, that the next step in constructing high-quality studies on the influence of vitamin D as a medicine in UFs therapy should be the use of the vitamin D response index when creating patient groups.

Since the correction of vitamin D concentrations has a positive effect on the inhibition of UF growth, we should also consider other therapies (except supplementation) which will increase serum vitamin D levels. Harmon et al. reported that the use of estrogen-containing contraceptives was associated with a 20% increase in serum 25(OH)D concentrations [143]. Our studies have also recently shown that the use of combined oral contraception (COC) with drospirenone results in higher serum vitamin D levels [144]. The exact mechanisms causing the increase are still unknown, so research should be continued. The use of selected form of oral contraception in selected groups could reduce UF-related symptoms as well as help to maintain the correct serum concentrations of vitamin D. It should be emphasized that COC use should not be expected to reduce the tumor volume [138]. In the short-term management, COC can be used to reduce menstrual bleeding associated with UFs [138]. It should be emphasized that according to available data COC use slightly increase the overall risk of breast [145] cancer [146] occurrence. On the other hand, this risk is counterbalanced by the lower risk of endometrial, ovarian, and colorectal cancer in the future in women who used the COC [147].

In light of the above, paricalcitol has a great potential to become an effective drug or co-drug for the conservative treatment of UFs [34]. Paricalcitol effectively reduces the proliferation of human leiomyoma cell cultures and fibroid tumor volumes, and induces apoptosis [105]. Further extensive clinical research is necessary to gain more information about the use of paricalcitol in UF therapy (Figure 3). In the meantime, other VDR analogs should be studied for their potential role in the management of UFs [141].

Due to rare side effects and relatively high safety of vitamin D, we could also consider combination therapies—drugs with additional simultaneous vitamin D supplementation. According to a very recent study by Ali et al., UPA and vitamin D share synergistic anti-fibroid activities [140]. In this study, the combined therapy of UPA and vitamin D resulted in a significant inhibition of UF cell growth (lowest proliferation rate from all studied groups) [140]. This research is a milestone and can bring entirely new perspectives on how to treat UF. We are of the opinion that such treatment would be beneficial in selected populations. Perhaps it could be more effective than the traditional approach in patients with the most severe symptoms, such as in obese African-Americans with vitamin D deficiency. It could also be treated as a type of add-back therapy during gonadotropin releasing hormone (GnRH) analog treatment [148], such as to prevent bone loss [149] or negative effect on mood or cognition [150] caused by estrogen deficiency. Similar studies performed on other substances such as GnRH analogs, for example, leuprolide, goserelin, elagolix or relugolix, might constitute the next step. If the safety of such therapies will be confirmed, the studies should be transferred to the next stages of clinical trials in humans.

Early prevention, appropriate prophylaxis, as well as treatment of UFs at an early stage in high-risk women, are priority actions. Perhaps the solution for the future will be to identify high-risk groups before the appearance of UFs, and then to implement preventive measures. The ideal methods of prevention and early-stage therapy should be inexpensive and relatively free of risk [13,42]. Highly individualized and personalized multi-drug therapies with the use of vitamin D might also be considered.

High-dose vitamin D and vitamin D analogs alone or as co-drugs can sooner or later become optimal, effective, safe drugs for conservative treatment of UFs. First, however, they must undergo advanced clinical trials, where they can confirm their effectiveness.

## 4. Conclusions

Vitamin D plays an essential role in UF biology. Vitamin D and its analogs seem to be promising, effective, and low-cost compounds in the management of UFs and their clinical symptoms. In cases of further positive observations and randomized control trials, vitamin D preparations could become new tools in the fight against UFs, with the additional beneficial pleiotropic effect. Further studies about the biological effect of vitamin D on UF biology are essential. The synergy between vitamin D and selected anti-UF drugs is a very interesting issue which requires further research.

## Abbreviations

1,25(OH)D	1,25-dihydroxyvitamin D
25(OH)D	25-hydroxyvitamin D
BMI	body mass index
CDK1	cyclin-dependent kinase 1
COC	combined oral contraception
COMT	catechol-*O*-methyltransferase
ECM	extracellular matrix
EMA	European Medicines Agency
FEN-1	flap structure-specific endonuclease 1
GnRH	Gonadotropin releasing hormone
MMP	matrix metalloproteinase
mTOR	mechanistic target of rapamycin
NHANES	National Health and Nutrition Examination Survey
PCNA	proliferating cell nuclear antigen
QoL	quality of life
SELF	Study of environment lifestyle and fibroids
SNP	single nucleotide polymorphism
SPRM	selective progesterone receptor modulator
TGF-β	transforming growth factor beta
TIMP	tissue inhibitor of metalloproteinase
UF	uterine fibroid
UPA	ulipristal acetate
VDBP	vitamin D binding protein
VDR	vitamin D receptor
